# Dynamics of the aortic annulus in 4D CT angiography for transcatheter aortic valve implantation patients

**DOI:** 10.1371/journal.pone.0184133

**Published:** 2017-09-08

**Authors:** Mustafa A. Elattar, Leon W. Vink, Martijn S. van Mourik, Jan Baan, Ed T. vanBavel, R. Nils Planken, Henk A. Marquering

**Affiliations:** 1 Department of Biomedical Engineering and Physics, Academic Medical Center, University of Amsterdam, Amsterdam, The Netherlands; 2 Department of Heart Center, Academic Medical Center, University of Amsterdam, Amsterdam, The Netherlands; 3 Department of Radiology, Academic Medical Center, University of Amsterdam, Amsterdam, The Netherlands; Universita degli Studi di Bologna, ITALY

## Abstract

**Background:**

Transcatheter aortic valve implantation (TAVI) is a well-established treatment for patients with severe aortic valve stenosis. This procedure requires pre-operative planning by assessment of aortic dimensions on CT Angiography (CTA). It is well-known that the aortic root dimensions vary over the heart cycle. However, sizing is commonly performed at either mid-systole or end-diastole only, which has resulted in an inadequate understanding of its full dynamic behavior.

**Study goal:**

We studied the variation in annulus measurements during the cardiac cycle and determined if this variation is dependent on the amount of calcification at the annulus.

**Methods:**

We measured and compared aortic root annular dimensions and calcium volume in CTA acquisitions at 10 cardiac cycle phases in 51 aortic stenosis patients. Sub-group analysis was performed based on the volume of calcium by splitting the population into mildly and severely calcified valves subgroups.

**Results:**

For most annulus measurements, the largest differences were found between 10% and 70 to 80% cardiac cycle phases. Mean difference (±standard deviation) in annular minimum diameter, maximum diameter, area, and aspect ratio between mid-systole and end-diastole phases were 1.0 ± 0.29 mm (p = 0.065), 0.30 ± 0.24 mm (p = 0.7), 24.1 ± 7.6 mm^2^ (p < 0.001), and 0.041 ± 0.012 (p = 0.039) respectively. Calcium volume measurements varied strongly during the cardiac cycle. The dynamic annulus area was behaving differently between mildly and severely calcified subgroups (p = 0.02). Furthermore, patients with severe aortic calcification were associated with larger annulus diameters.

**Conclusion:**

There is a significant variation of annulus area and calcium volume measurement during the cardiac cycle. In our measurements, only the dynamic variation of the annulus area is dependent on the severity of the aortic calcification. For TAVI candidates, the annulus area is significantly larger in mid-systole compared to end-diastole.

## Introduction

Aortic valve stenosis (AS) has a strong age-associated prevalence of 0.2% in adults between 50–59 years, increasing to 9.8% for the age of 80–89 years [1,2]. Approximately one-third of all patients with severe aortic stenosis are not eligible for surgery, mainly because of high age, left ventricular dysfunction, or other significant co-morbidities [3,4].

Transcatheter aortic valve implantation (TAVI) has been introduced as an alternative treatment for these high-risk patients providing sustained clinical and hemodynamic benefits [4–6]. Preprocedural evaluation of the aortic root sizing parameters, especially aortic annular dimensions, is pivotal for selecting the optimal prosthesis size [7–10]. CT Angiography (CTA) imaging plays an important role in pre-operative interventional planning and patient selection [11–13].

In clinical practice, aortic root dimensions are typically determined by CTA at either of two points in the cardiac cycle [14,15]. Some use mid-systole, where the valve is open to facilitate the annular insertion point identification [16]. Others prefer end-diastole, at 75% of the R-R interval, when the aortic valve movement is minimal and the motion artifacts are reduced, which makes the analysis easier and more robust [17].

Studies in animal models and healthy humans have shown annulus diameter variations between mid-systole and end-diastole of as much as 7.5% [18–21]. Such variations seem relevant for prosthesis selection. Yet, it is not clear whether similar dynamic variations in annular dimensions exist in elderly patients with calcified aortic stenosis (AS) in whom aortic compliance is likely to be substantially reduced. Earlier studies on dynamic changes in annular dimensions in the context of TAVI have produced conflicting results [9,22,23]. In addition, these studies were limited to two time points, mid-systole and end-diastole. Yet, aortic dimensions change continuously during the cardiac cycle, and measuring differences between only these time points may underestimate the complexity of the dynamics [9]. A recent study assessed aortic root dimensions along the cardiac cycle to find the largest diameter measurements in TAVI populations [24]. This study showed that the mid systole is featuring the largest dimensions, although the effect of annulus calcification severity on the annulus dynamics was not studied.

Calcification of the annulus is highly relevant for TAVI planning, since it is strongly associated and a good predictor for post procedural prosthesis eccentricity and paravalvular regurgitation [25–27]. Furthermore, the elasticity of the aortic annulus is dependent on the extent of calcium deposit [28]. Because of the large density differences between calcifications and its surroundings and the limited spatial and temporal resolution, it is expected that calcium volume measurements on CT imaging are susceptible to motion related artifacts. The movement of high-density structures during imaging influences its representation in CT images. However, no previous studies have evaluated the dynamic variation of calcium scoring during the cardiac cycle. Severity of calcium at the annulus level may increase the partial volume averaging effect (blooming) altering the quality of the annulus sizing [10].

The purpose of the current study is to evaluate dynamic variations in annulus dimensions and calcium score in patients with aortic stenosis. In addition, we assess the effect of calcification severity on the annulus sizing measurements.

## Methods

### Study population

The study included consecutive 53 patients with severe symptomatic aortic stenosis who were referred to our institute (Academic Medical Center, The Netherlands) for TAVI who underwent preprocedural ECG-gated cardiac 4D CTA. The Academic Medical Center (University of Amsterdam) medical ethics committee granted approval of the study design and waived informed consent since solely data obtained in the context of clinical care was utilized. Patients with permanent pacemaker, coronary artery bypass grafting, bicuspid valves, lack of calcification, and aortic root dilation were excluded from the study. As a result, we excluded one patient with a bicuspid valve and one patient without calcification resulting in a study population of 51 patients. The population consisted of 27 [53%] female and 24 males with an average age of 82 ± 7 years. Other patient characteristics for TAVI candidate patients are described in Table 1.

**Table 1:** Baseline characteristics for the included patient cohort.

Characteristics		Result
Number of patients		51
Gender, female		27 (53%)
Age (years)		82 (±7)
Height (cm)		168 (±9)
Weight (kg)		74.6 (±13)
Body Mass Index (kg/m^2^)		26.4 (±4.5)
Body Surface Area (m^2^)		1.86 (±0.2)
Left Ventricle Ejection Fraction (Echo)		
Good	≥ 55%	31 (61%)
Mildly Impaired	45%–54%	8 (15%)
Moderately Impaired	30%–44%	10 (20%)
Poor	< 30%	2 (4%)

Data presented as mean (±SD) or numbers (percentages).

### Scan protocol

All CT-scans were performed on a Philips Brilliance 64 slice CT scanner; imaging parameters were 120 kV, matrix 512, and convolution kernel B. The chest, abdomen, and pelvis were scanned using one bolus of 120 ml contrast Iomeron 400, intravenously infused at a rate of 5 ml/s. Image volumes contained 500 to 600 slices. The dimensions of each slice were 512 × 512 pixels with a 16-bit depth. The in-plane image resolution was isotropic and varied between 0.44 mm and 0.68 mm. The slice thickness for all data sets was 0.9 mm with an overlap of 0.45 mm. The 4D CTA scanning protocol produced successive 10 cardiac cycle phase volumes covering the full cardiac cycle (each 10% of RR-interval)

### Aortic root analysis

**Fig 1:**
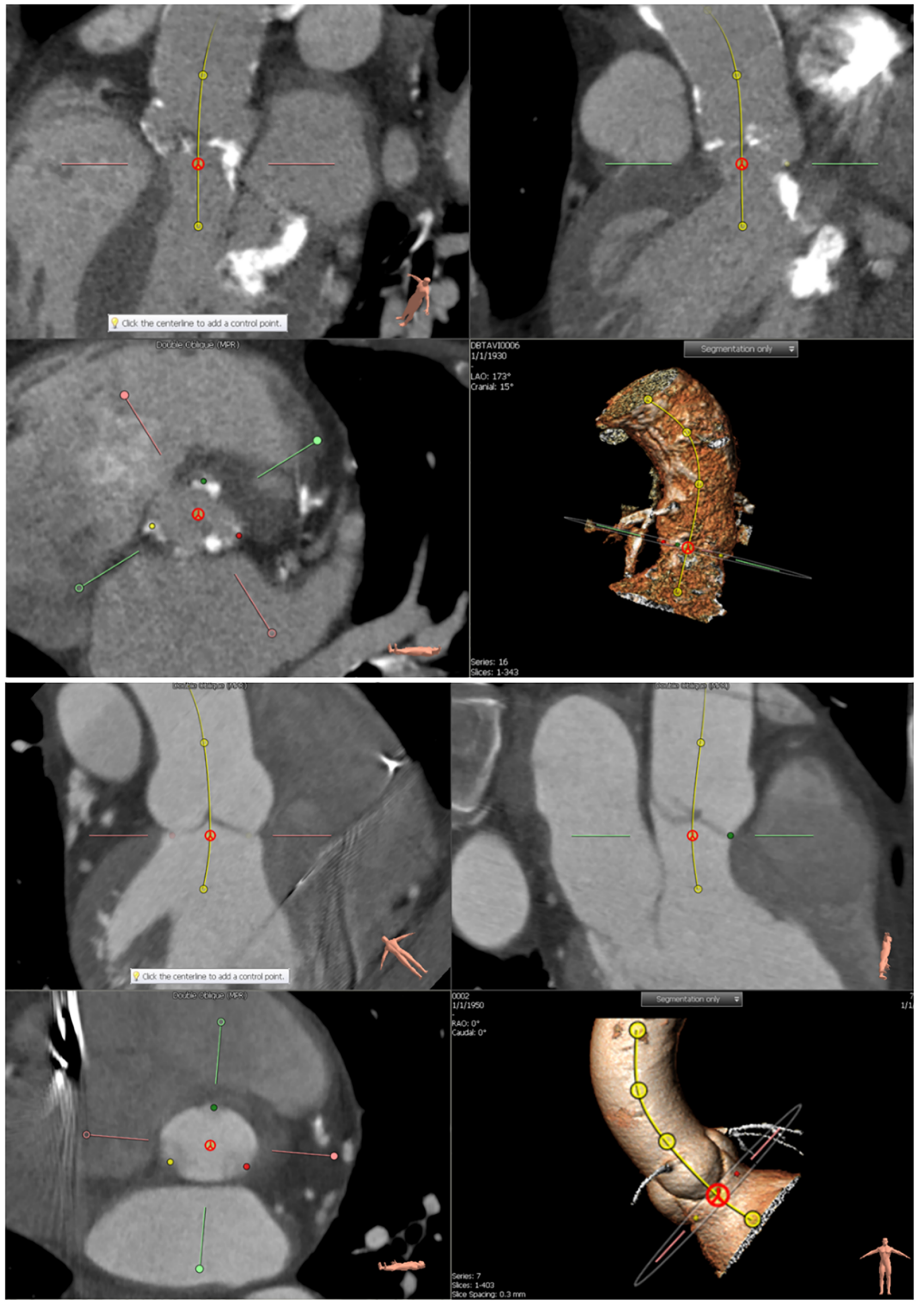
Two examples of the annulus plane refinement (TAVI candidate and Non-calcified aortic valve). Three MPR images and a volume rendering image are shown. The upper frames show the vessel view including the centerline. The bottom left pane shows an image perpendicular to the centerline at the annulus plane. In this frame the three hinge points in red, green, and yellow are shown. The bottom right view shows a 3D rendered view for the aortic root and the centerline.

Aortic root measurements were performed by two independent observers using the 3mensio Valves software (version 5.1; Pie Medical Imaging, Maastricht, The Netherlands) [29,30]. Rater 1 analyzed 22 subjects and rater 2 analyzed 34 subjects with an intersection of five subjects for the interobserver variability assessment. Both raters are trained biomedical engineers with a specialization in CT imaging and image analysis for TAVI candidate patients. The training was performed by cardiovascular radiologist and interventional cardiologist (both with > 10 years of experience). Each cardiac cycle phase volume was loaded after which the centerline of the aortic root was automatically detected. Subsequently, the observer could adjust the aortic root centerline by refining centerline control points, resulting in an adjusted multi-planar reconstruction images (MPRs). In the next step, the software automatically defined the annulus plane that lies between the aortic root and the left ventricle outflow tract. It was manually adjusted to assure that the annulus plane included the three hinge points. The hinge points correspond to the three anchors at the nadir of each of the attachments of the aortic cusps [31]. The annulus plane adjustment was performed on two oblique MPRs and the perpendicular plane to the initial centerline (Fig 1).

After confirmation of the annulus plane, two oblique stretched vessel views were generated showing the left ventricle outflow tract (LVOT), aortic valve, and ascending aorta along the centerline. This oblique stretched vessel view facilitated the manual hinge point selection. In this manner, the selected annulus plane was assured to include the three hinge points of the annulus.

**Fig 2:**
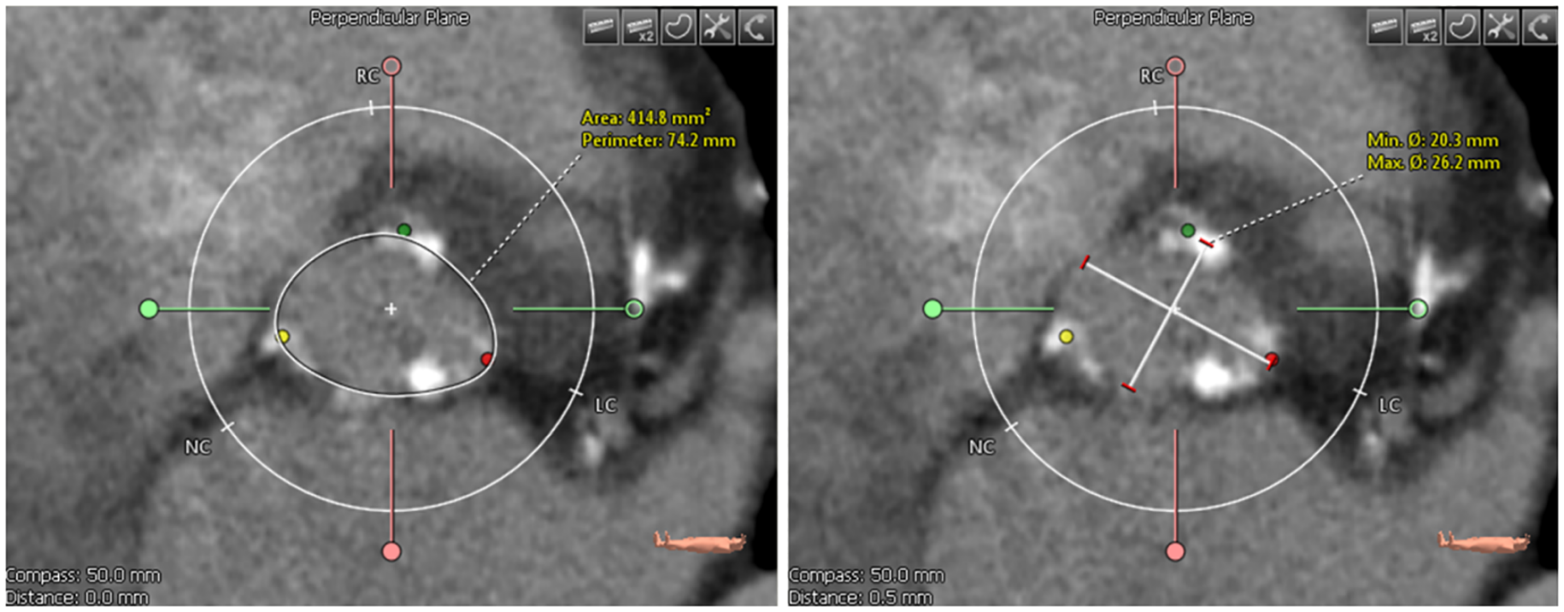
(Left) Annulus image showing a drawn polygon with the calculated area and perimeter. (Right) Annulus image showing the drawn minimum and maximum diameter.

Annulus area and perimeter were determined after drawing a polygon along the aortic annulus edge as shown in Fig 2 on the annulus plane. The polygon was drawn following the annulus wall even if there was calcification; the polygon line was drawn through the calcium deposits sticking to the vessel wall. On the same plane, minimum and maximum diameters were measured using a straight-line annotation tool. Annulus perimeter was annotated only by rater 2 resulting in 32 patients after applying exclusion criteria. The annulus aspect ratio was calculated by dividing minimum diameter by the maximum diameter.

**Fig 3:**
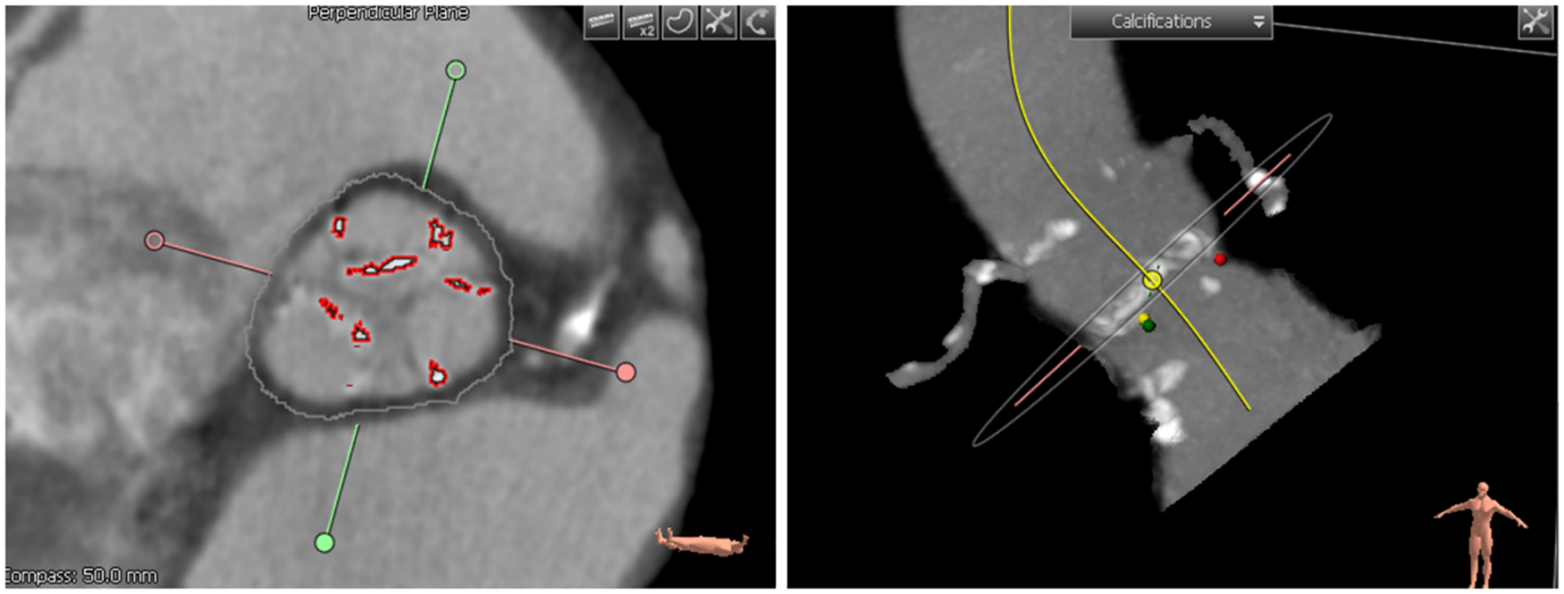
(Left) Aortic root cross-section image showing extracted calcifications as depicted by red contours. (Right) 3D rendered maximum intensity projection view of the aortic root with calcifications displayed in white.

A calcium scoring tool was used to assess the calcification volume after choosing the threshold in Hounsfield Units (HU) that separates the calcification from the contrast-enhanced blood and aortic wall intensities (Fig 3). Calcification volume was calculated after setting a volume of interest (VOI) including aortic annulus and leaflet calcifications. This VOI was set to exclude calcifications in the LVOT, coronary arteries, and ascending aorta. Subsequently, raters determined the proximal and distal cutting planes such that these areas were excluded. If there were still other undesirable calcium deposits within the VOI, a contour editing tool was used to exclude extra deposits.

Within a single patient, the same calcium threshold and VOI were used for all cardiac cycle phases. For the interobserver analysis, calcium threshold values and cutting planes were identical for every patient for both raters after consensus.

### Calcium subgroup analysis

Two subgroups were generated based on the amount of annulus and leaflet calcification. Patients with calcification volumes exceeding 250 mm^3^ were assigned to the severely calcified patient group. The mildly calcified group included patients with calcification volumes less than 250 mm^3^. After visual inspection, the histogram suggested a bi-model Gaussian distribution. The fitting of the probability distribution of a Bi-Model Gaussian with the histogram of the calcium volume measures, resulted in a threshold of 250 mm^3^ according to the model components means of 136 and 349 mm3.

To study if there will be different annulus dynamics between mild and severe calcifications subgroups, we compared both subgroups annulus dynamics. The dynamic variation of the calcium volume measurement and geometric measures in a single patient is presented as the range and the standard deviation of the measures of the ten cardiac phases. We determined the extent of all ranges by subtracting the minimal value from the maximal value of the measures. The mean and medians of the single-patient standard deviation and extents for each subgroup were compared. The variation parameters incorporated standard deviation and range for the annulus measurements during the cardiac cycle for each patient. Mean variation per annulus measurements and the significance numbers were reported.

### Statistical analysis

Continuous data that follow normal distribution are presented as mean ± standard deviation (SD). Statistical analyses were performed with MATLAB (Version R2013b, The Mathworks Inc., and Natick, MA) and SPSS (version 19.0, SPSS Inc., and Chicago, IL). We also reported the average of all measurements per each subgroup created based on the amount of calcification (lower and higher than 250mm^3^).

Significance of differences of annulus measurements (diameters, aspect ratios, areas, and perimeters) between the cardiac cycle phases was determined by paired t-tests for normally distributed data and Kolmogorov-Smirnov tests for data that did not follow normal distribution. Aortic annulus measures were compared between all cardiac phases. P-value matrix plots were used to visualize all comparisons between the measurements of the various cardiac cycle phases. Differences with p-values of 0.05 or less were considered statistically significant.

Since annulus area, perimeter, and calcification volume measurement variation ranges were widely different between patients (see Table 2), we normalized them. For every patient independently, the measures were normalized by offsetting the value by the minimum value of all cardiac phases, and dividing the resulting value by the range (maximum-minimum difference) of measures. As a result, these measures were scaled to fall between 0 and 1 for each patient.

**Table 2:** Mean and standard deviation of annulus measurements (minimum, maximum diameter, annulus aspect ratio, area, and perimeter) and aortic annulus calcification volume. Mid-systole and end-diastole phases are gray highlighted in the table.

Cardiac cycle phase	Minimum Diameter (mm)	Maximum Diameter (mm)	Annulus Aspect Ratio	Area (mm^2^)	Perimeter (mm)	Calcium Volume (mm^3^)
	Mean ± STD (n = 51)	Mean ± STD (n = 51)	Mean ± STD (n = 51)	Mean ± STD (n = 51)	Mean ± STD (n = 32)	Mean ± STD (n = 51)
0%	23.6 ± 2.7	29.3 ± 3.1	0.81 ± 0.07	548 ± 107	83.8 ± 8.9	325 ± 249
10%	24.3 ± 2.7	29.3 ± 2.9	0.83 ± 0.07	558 ± 103	84.2 ± 8.0	286 ± 228
20%	24.3 ± 2.9	29.3 ± 2.7	0.83 ± 0.08	559 ± 108	84.6 ± 8.9	291 ± 236
30%	24.2 ± 3.0	29.4 ± 2.9	0.83 ± 0.06	559 ± 108	84.6 ± 8.5	315 ± 229
40%	23.6 ± 2.9	29.2 ± 3.1	0.81 ± 0.07	544 ± 111	83.6 ± 9.1	337 ± 251
50%	23.4 ± 2.8	29.2 ± 3.2	0.80 ± 0.07	533 ± 111	83.4 ± 8.7	306 ± 236
60%	23.4 ± 2.7	29.2 ± 3.0	0.80 ± 0.07	538 ± 114	83.5 ± 9.0	305 ± 232
70%	23.2 ± 2.6	29.2 ± 2.8	0.79 ± 0.07	535 ± 106	82.5 ± 8.2	319 ± 227
80%	23.1 ± 2.4	29.1 ± 3.2	0.79 ± 0.07	534 ± 105	83.1 ± 8.8	320 ± 245
90%	23.1 ± 2.9	28.9 ± 2.9	0.80 ± 0.08	537 ± 113	82.8 ± 9.1	307 ± 231

### Interobserver variability

Five arbitrarily selected patients were evaluated by two raters resulting in 100 measured minimum and maximum diameters. For these, scatter plots were generated and the intraclass correlation coefficient was calculated. Inter-observer variability was furthermore assessed by Bland and Altman analysis.

## Results

The aortic annulus measurements in the ten cardiac phases are presented in Table 2. Mean difference (± standard deviation) between mid-systole and end-diastole phases were for annulus area 24.1 ± 7.6 mm^2^ (p < 0.001), annulus perimeter 2.1 ± 0.8 mm (p < 0.001), annulus minimum diameter 1.0 ± 0.29 mm (p = 0.065), annulus maximum diameter 0.30 ± 0.24 mm (p = 0.74), annulus aspect ratio 0.041 ± 0.012 mm (p = 0.039), and annulus calcium volume 4.12 ± 74.9 mm^3^ (p = 0.93).

**Fig 4:**
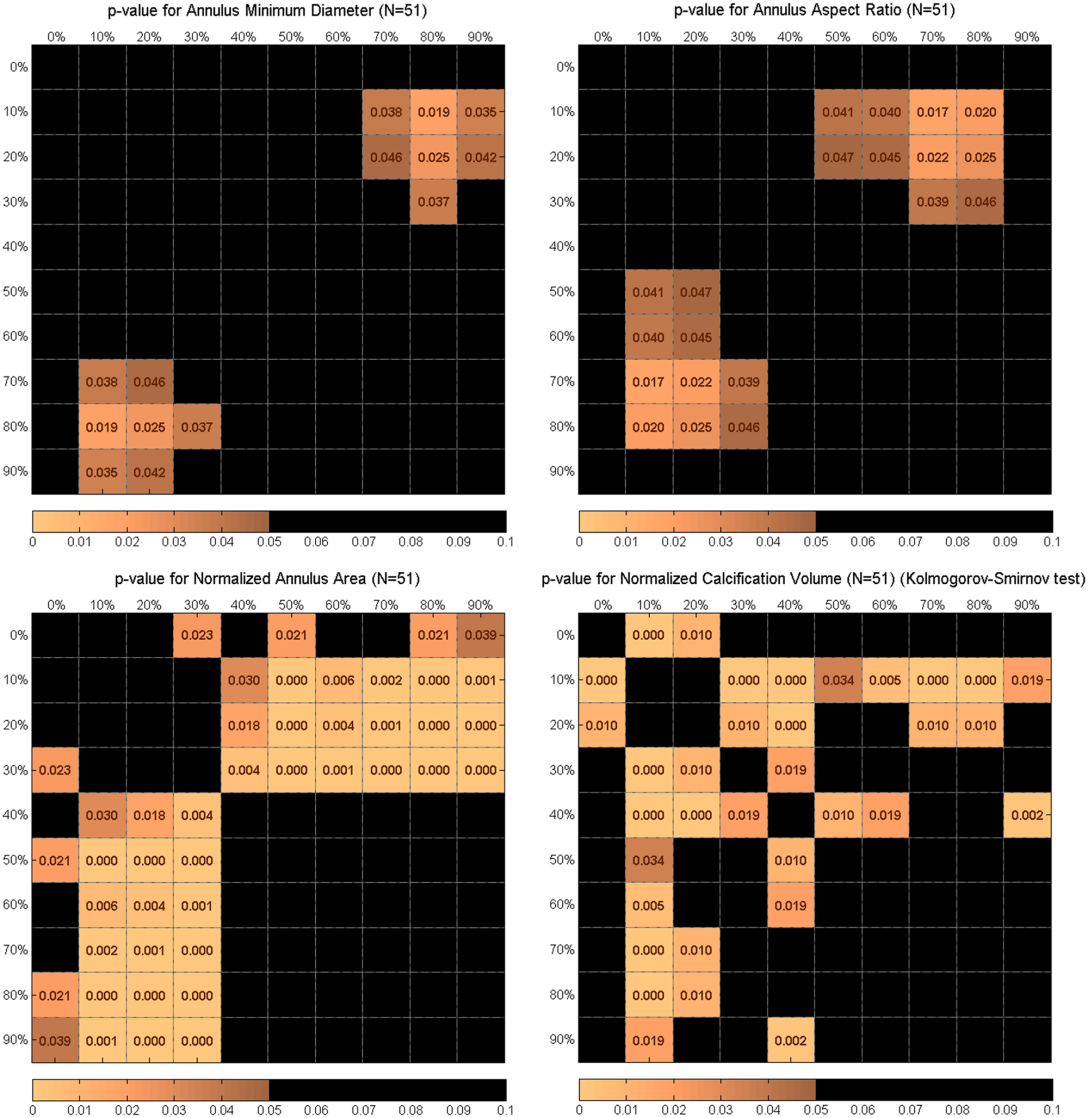
P-value matrices showing statistically significant difference between all combinations of cardiac cycle phases for annulus minimum diameter, annulus aspect ratio, normalized annulus area, and normalized calcifications volume.

Aortic annulus area was significantly different between mid-systole and end-diastole (p = 0.001) with areas of 559 ± 108 mm^2^ and 535 ± 106 mm^2^ respectively. This difference in area was 4.5% the two time phases for the absolute area difference and 25% for the normalized annulus area. Cardiac cycle phases of 10% to 30% showed significant differences with all cardiac cycle phases between 40% and 90% as shown in Fig 4. The annulus perimeter dynamic behavior was similar to the annulus area during the cardiac cycle where average perimeter at mid-systole was 84.6 ± 8.5 mm and at end-diastole it was 82.5 ± 8.2 mm. The differences between mid-systole and end-diastole for the mean minimum and maximum annulus diameters were not statistically significant with p-values of 0.065 and 0.74, respectively. Fig 4, which shows differences between all cardiac phases, shows statistically significant differences in aortic root measurements for various phase combinations.

The annulus aspect ratio was 0.83 ± 0.06 and 0.79 ± 0.07 for mid-systole and end-diastole, respectively, which was statistically significant different (p = 0.04).

Annulus area, minimum diameter, aspect ratio, and showed similar dynamic characteristics through the cardiac cycle, with increasing values at mid-systole and reaching minimum values at late diastole.

The average and standard deviation of threshold to separate calcified tissue was 650 ± 70 HU. There was no significant difference in annular calcium volume measurement between end-diastole and mid-systole measurements (p = 0.69). However, there was a statistical significant difference in calcium volume measurement between 10% and 40% with an average calcium volume of 286 mm^3^ and 337 mm^3^, respectively (p < 0.001) (Fig 4).

Average measurement variation per patient was found to be significantly different between subgroups for only normalized annulus area (p< 0.001) and there was no difference for other measurements between subgroups (Table 3).

**Table 3:** Average dynamic variation of annulus measurements per cardiac cycle for both subgroups (mildly annulus calcified subgroup (≤ 250 mm3) and severely annulus calcified subgroup (> 250 mm3)). Annulus measurements included are minimum diameter, maximum diameter, annulus aspect ratio, normalized annulus area, normalized calcium volume, annulus area, and calcium volume. P-values for significant subgroup difference for dynamic variation and absolute measurements are shown in the last two rows. Severely annulus calcified subgroup is gray highlighted in the table.

	Average Temporal Measures	Minimum Diameter (mm)	Maximum Diameter (mm)	Annulus Aspect Ratio	Normalized Annulus Area	Normalized Calcium Volume	Annulus Area (mm^2^)	Calcium Volume (mm^3^)
Mildly Annulus Calcified Group (< 250 mm3)	Mean	22.8	28.5	0.80	0.48	0.52	512	127
	STD	1.40	1.08	0.06	0.36	0.33	37.4	29.8
	Range	4.36	3.42	0.18	-	-	104	88.9
	Minimum	20.8	26.9	0.72	-	-	461	80.9
	Maximum	25.1	30.3	0.90	-	-	565	170
Severely Annulus Calcified Group (> 250 mm3)	Mean	24.4	29.9	0.82	0.47	0.57	576	488
	STD	1.22	1.32	0.05	0.31	0.33	32.3	58.3
	Range	3.75	4.34	0.17	-	-	107	177
	Minimum	22.7	27.7	0.74	-	-	526	388
	Maximum	26.5	32.0	0.91	-	-	632	565
Groups Dynamic Difference P-value		(0.22)	(0.09)	(0.59)	(< 0.001)	(0.74)	(0.09)	(<0.001)
Groups Difference P-value		(< 0.001)	(< 0.001)	(0.02)	(0.02)	(<0.001)	(<0.001)	(<0.001)

**Fig 5:**
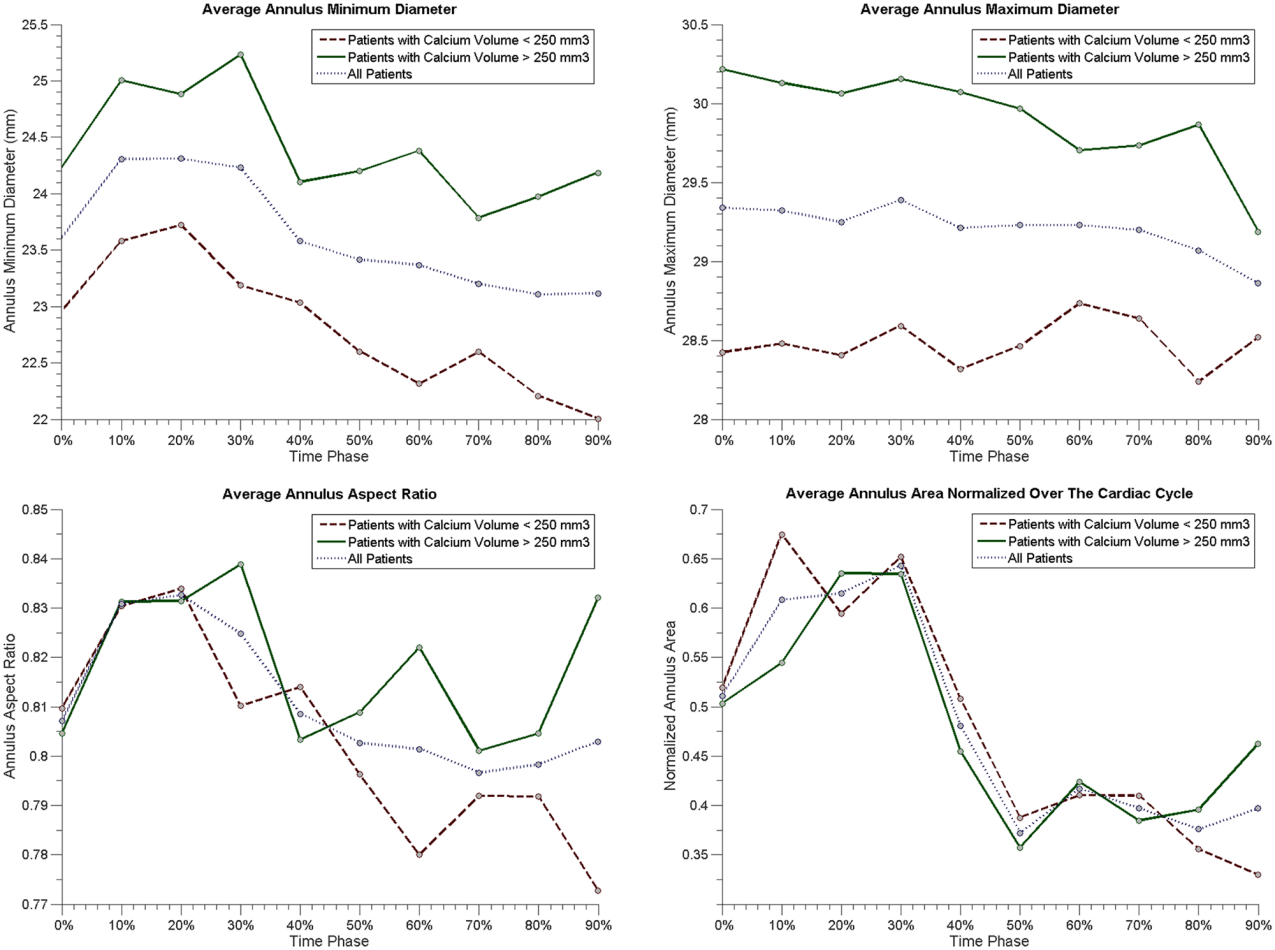
Average annulus minimum diameter, maximum diameter, aspect ratio, and area for all patients (dotted blue line), subgroup 1 with patients with calcium volume less than 250 mm3 (dashed red line), and subgroup 2 for calcium volume exceeding 250 mm3 (solid green line).

However, the average measurements were different between subgroups for minimum and maximum diameters (both p< 0.001) which can be observed in Fig 5.

**Fig 6:**
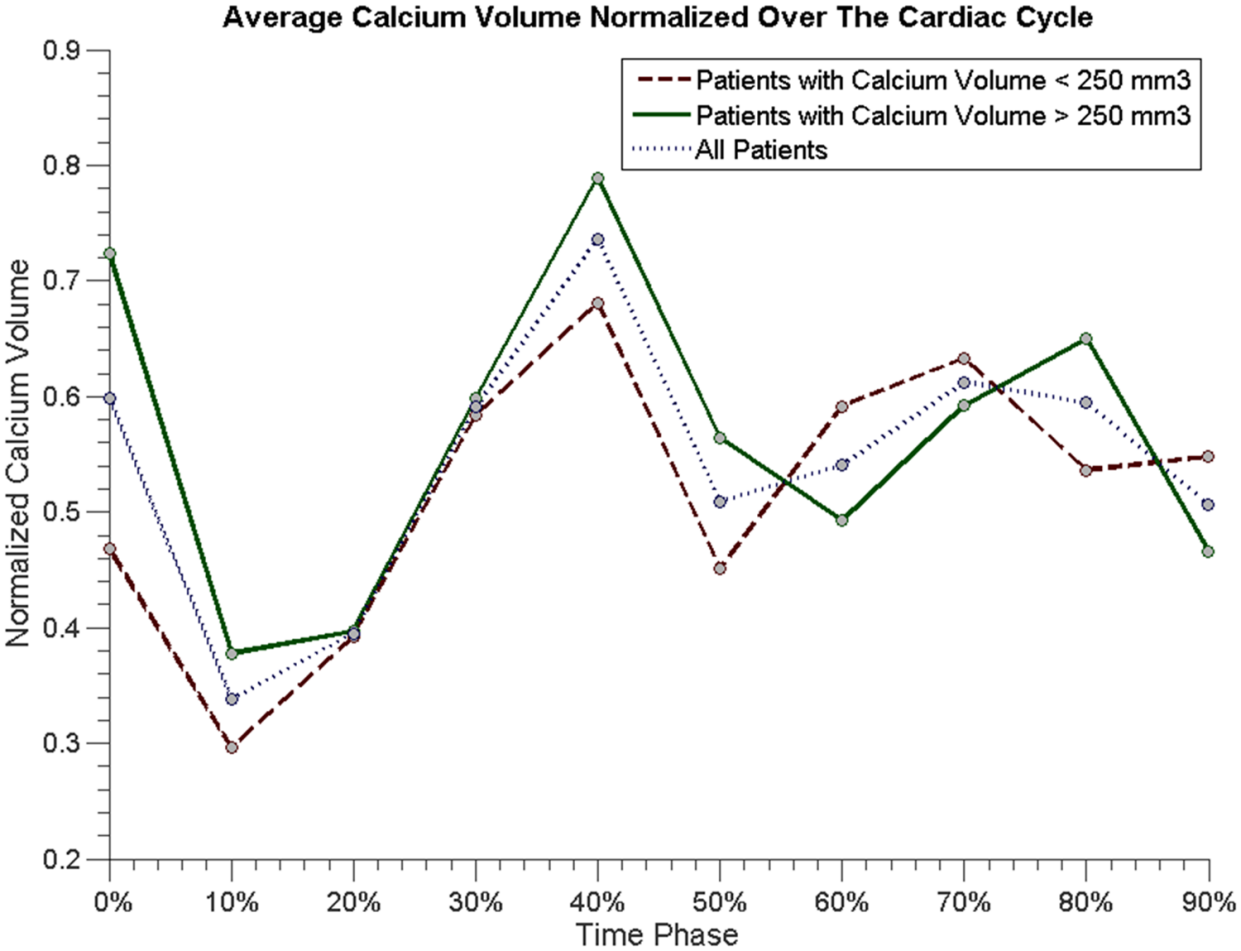
Average normalized calcium volume for all patients (dotted blue line), patients with calcium volume lower than 250 mm3 (dashed red line), and calcium volume exceeding 250 mm3 (solid green line).

Average minimum, maximum diameter, annulus aspect ratio, and area for the heavily and mildly calcified populations are shown in Fig 5. In Fig 6, average normalized calcification volume is presented for the calcification subgroups and the full population combined.

**Fig 7:**
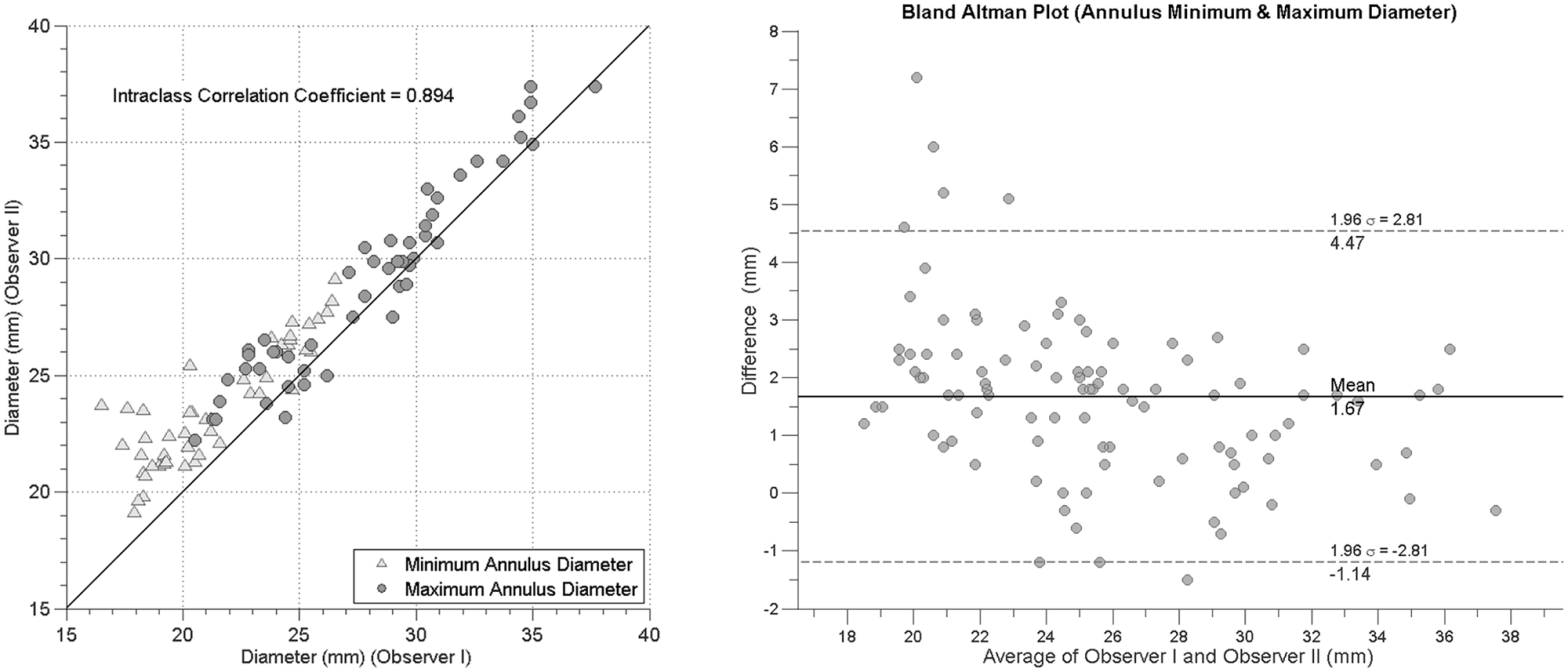
(Left) Scatter plot of annulus diameter measurements of rater 1 versus rater 2. (Right) Bland–Altman plot of the agreement between rater 1 and rater 2 for the annulus minimum and maximum diameters.

The agreement between rater measurements is illustrated in a scatter plot in Fig 7. Intraclass correlation coefficient (ICC) showed strong agreement (ρ = 0.89) between raters for both minimum and maximum diameters. Bland–Altman analyses showed a mean paired difference of 1.67 mm and standard deviation of 1.9 mm for the diameter measurements (Fig 7).

## Discussion

This study shows significant dynamic variations in aortic annular area, perimeter, minimum diameter, and aspect ratio during the cardiac cycle in TAVI-eligible patients. Only the annulus area, perimeter, and aspect ratio were significantly different between systolic and diastolic phases. Annular minimum and maximum diameters did not show a difference between systolic and diastolic phases. However, a significant difference in annular minimum diameter was found between the 30% and 80% phases. We found large differences exceeding 33% in the dynamic calcium volume measurements. The smallest volumes were around the 10% cardiac phase and the largest volumes around the 90% of the cardiac phase.

The dynamic behavior of the annulus area was significantly different along the cardiac cycle between the severely and mildly calcified subgroups. Severely calcified patients were found to have significantly larger minimum and maximum diameters in comparison with the patients with mildly calcified aorta annulus and leaflets for all cardiac cycle phases. The calcification measurements have shown low values especially at the 10% of R-R interval and higher at the 40% of R-R interval. The largest difference was found to be between these two cardiac cycle phases with about 38% change in volume on the normalized scale.

Anatomical changes in the aortic annulus and aortic root between mid-systole and end-diastole have been suggested by earlier experimental and imaging studies [5, 6]. A previous study using transthoracic echocardiography of 292 patients data suggested significant aortic annular diameter changes with a mean change of 7.5% [20]. This is in line with the distensible annulus behavior in other studies who studied the dynamic behavior of the aorta in patients with and without aortic stenosis [9,18,19,32]. However, in our study this effect was not observed (between end-diastole and mid-systole). It should be noted that in the study of Yankah et al, the majority of patients were free from aortic stenosis (60%) [20]. This could explain the smaller differences found in our study with changes of minimum and maximum diameters between mid-systole and end-diastole of 4% and 0.8% respectively.

Another two studies on CTA, showed no significant differences in annulus dimensions between mid-systole and end-diastole. In [33], only 25 patients data free from aortic stenosis was used, where in [23] 52 patients with aortic calcification were analyzed. However, in these studies only measurement differences between mid-systole and end-diastole was assessed. None of the mentioned studies have assessed the dynamic behavior in cardiac cycle phases other than mid-systole and end-diastole.

In a recent study, Jurencak et al. also concluded that within a heart cycle there were significant differences in most aortic root measures. They also observed that the maximum diameter was not significantly changing. However, in this work [24] calcium measures and the effect of calcium severity, which is common in TAVI populations, on the annulus measurements were not studied.

We observed that the aspect ratio of the annulus lumen increases during diastolic phases and decreases during the systolic phases (10% to 30%). The largest difference was between 10% and 70%, which confirms previous findings [24,34] that the annulus remains elliptical during the whole cardiac cycle.

Maximum annulus diameter measures between mid-systole and end-diastole were found to be similar, and this can be explained by the relatively old patients in our TAVI cohort. Older patients usually suffer from higher levels of aortic wall stiffness due to alteration in collagen fibers that makes the aortic wall tending to be thicker and denser [35,36], which results in a reduction of the distensibility of the annulus.

The annulus diameters were found to be larger in patients with a severely calcified annulus. We believe that annular calcifications may have biased the measurement because of calcium blooming effect leading to an over estimation of the diameters.

The aortic valve opens between 10% and 40% phases. In this time window, leaflet motion is maximal. This contributes to motion artifacts, which in CT are expected to cause blurring of the calcium. This blurring may exaggerate the actual calcium volume. This agrees with our findings of having the maximum difference in calcium volume between the 10% and 40% phases.

To the best of our knowledge, this is the first study that studied the dynamic behavior of the annulus anatomical measurements over the full cardiac cycle and in relation to annulus calcifications in TAVI candidates. Full cardiac cycle measurements allowed us to generate p-value matrices that show the significance of differences in measurements for different cardiac cycle phases. We believe that both presenting all combinations of time frames with the P-matrices and discussing the results for commonly clinically used cardiac phases is optimal for this study.

This is a single center retrospective patient study including a relatively small number of patients, which may not be a representative sample of the wider TAVI population. The number of patients and the number of raters that was used for the interobserver variability analysis was small. Also, there was a small bias between the two observers. Further analysis is needed to confirm the dynamic behavior observed in this study. Some measures were normalized because of large inter-patient differences e.g. area, perimeter, and calcium volume. This normalization may have reduced the statistical significance. We used calcium volume for subgroup analysis in which we did not correct for the aortic root volume. It could be expected that larger root volumes may have larger calcium volumes. In future studies, the effect of relative vs absolute calcium volumes should be studied.

It is well known that the aortic root is distensible. Still, only mid-systole and end-diastole cardiac phases are used for aortic root sizing in clinical practice. We actually found that the largest and smallest measures were at other time phases than mid-systole and end-diastole. Therefore, it would be interesting to study the dynamic behavior in TAVI candidates with ruptured aortas, migrating valve stents, and left bundle branch block (LBBB) since the measurement at one phase might not represent the variation of the aortic annulus.

We found that in mid-systole, the aortic annulus area was larger than measured at end-diastole. This difference may influence the size selection. Therefore, the cardiac phase of measurement should be taken into consideration during planning. Surprisingly, this difference was not observed for the annulus diameter measurements.

Valve size selection guidelines suggest specific range of the annulus area for each valve size [37,38]. There is an overlap of 6 mm^2^ between these ranges for different valve sizes. Since the variation in dynamic annulus area measures is approximately four times as large as this value, is expected that these differences may affect the selection of the valve size of currently used TAVI valves in clinical practice.

This study suggests that annulus area measurements should be performed at the end-diastole phase. With the minor differences found in annulus diameters, this study suggests that for TAVI sizing there is little added value in having multiple cardiac phase image reconstructions. Calcium volume analysis suggests that calcium volumes should be performed at 10% of the cardiac cycle because for this phase the measured calcium volume is minimal. The volume is smaller at this phase because a reduction of the movement artifacts.

## Conclusion

There is variation of annular anatomical and calcium measurements along the cardiac cycle. The dynamic variation of the annulus area was depending on the severity of the aorta calcification. Variations of the measured calcium volumes may be as large as 48%. Small or no differences in minimum and maximum annulus diameters were found between mid-systole and end-diastole.

## Supporting information

S1 File Collected measurements. Sheet that include all collected measurements from all study patients.
(XLSX)
